# Ovarian fibroma as a novel indicator for burden of basal cell carcinoma in women with Gorlin syndrome: a retrospective cross-sectional analysis of the Gorlin syndrome national patient registry

**DOI:** 10.1097/JW9.0000000000000020

**Published:** 2022-06-15

**Authors:** Tiffany T. Wu, Deeti J. Pithadia, Joyce M.C. Teng

**Affiliations:** Department of Dermatology, Stanford University, California

**Keywords:** basal cell carcinoma, genetic counseling, Gorlin syndrome, nevoid basal cell carcinoma syndrome, ovarian fibroma, tumor suppressor gene

**What is known about this subject in regard to women and their families?**

- There is a wide spectrum of disease severity in Gorlin syndrome (GS) patients.
- Some females with GS develop ovarian fibroma (OF), which may significantly impact quality of life by causing frequent urination, abdominal pain, and ovarian torsion.
- Few risk factors for cutaneous disease burden in GS have been identified. What is new from this article as messages for women and their families?
- GS females who have OF also appear to have more severe cutaneous disease burden, thus requiring closer dermatological monitoring and potentially warranting early initiation of systemic therapy.

## Dear Editors,

Gorlin syndrome (GS), or nevoid basal cell carcinoma syndrome, is caused by pathogenic variant in the *PTCH1* tumor suppressor gene and characterized by basal cell carcinomas (BCCs), keratocystic odontogenic tumors of the jaw, medulloblastomas, and other cutaneous and skeletal features. GS is typically inherited but may be caused by a de novo mutation during development in 20 to 30% of cases.^1^ During their premenopausal years, affected women may develop ovarian fibromas (OFs), causing frequent urination, abdominal pain, and ovarian torsion. We aimed to evaluate the correlation of OF with cutaneous disease burden in females with GS.

A retrospective cross-sectional analysis was conducted using data from the national patient registry established by Gorlin Syndrome Alliance and Stanford University, which contains patient demographics, disease manifestations, and therapeutics received. Participants met clinical diagnostic criteria^2^ and/or were genetically confirmed.

The registry consisted of 202 adults. Of 116 females, 51 (44.0%) reported OF (Table 1). The proportion of women with high BCC burden (250+ BCCs) was significantly greater in the OF group (*P* = .025) (Table 2). Median age at evaluation was comparable among cohorts. Median age of diagnosis was younger in the OF group (14 vs 18 years, Table 1). Jaw cysts, a common tumorigenic manifestation utilized as control, had no correlation with BCC burden (*P* = .624) (Table 2). The proportion of women with OF on vismodegib, a hedgehog signaling inhibitor used to treat aggressive cutaneous disease, was significantly higher than in women without OF (58.3% vs 33.3%, *P* = .009) (Table 1).

**Table 1.** Demographics of adult patients participating in the Gorlin syndrome national patient registry established by Gorlin Syndrome Alliance and Stanford University

Adult patients (n = 202)		
Gender		
Females	125 (61.9%)	
Males	**77** (38.1%)	
Median age (range)		
Females with OF	54 years (19–75)	
Females without OF	56 years (19–80)	
Males	55 years (18–83)	
Median age of diagnosis		
Females with OF	14 years	
Females without OF	18 years	
Clinical manifestations		
Ovarian fibroma (females, n = 116)	51 (44.0%)	
Jaw cysts (n = 202)	92 (45.5%)	
Treatments		
Females with OF on systemic therapy	28 (58.3%)	*P* = .009^a^
Females without OF on systemic therapy	20 (33.3%)	

a *P* value represents Chi-square analysis examining proportion of patients on systemic therapy (vismodegib) in females with OF vs females without OF. The *P* value is statistically significant at a threshold of .05.

**Table 2.** Lifetime number of BCCs in association Gorlin syndrome-associated tumors

	Lifetime BCCs^a^		
	<10	250+	
OF			
OF	3	23	*P* = .025^b^
No OF	10	16	
Jaw cysts			
Jaw cyst	7	24	*P* = .624^b^
No jaw cyst	6	15	

BCC, basal cell carcinoma; OF, ovarian fibroma.

a Number of female patients who reported either less than 10 or greater than 250 lifetime BCCs.

b *P* values represent Chi-square tests examining proportion of patients in each group. Patients with 10 to 250 BCCs were excluded from these analyses. *P* values are statistically significant at a threshold of 0.05.

Our results demonstrate that women with OF have higher BCC burden are diagnosed younger and are more likely to be on systemic therapy. This association between OF and increased BCCs appears to be specific, rather than a general reflection of increased tumorigenesis. We theorize patients with more deleterious mutations^3^ or those with de novo mutations occurring earlier in embryogenesis may be more likely to develop OF and greater BCC severity.

GS is a clinically heterogeneous disorder, with lifetime number of BCCs varying from less than 10 to over 2000.^4^ The wide spectrum of phenotypic severity in GS highlights the importance of risk stratification. Besides radiation, UV exposure, and immunosuppression, other predictors of disease burden in GS are not well-established. Our study demonstrates OFs may be an important surrogate marker for BCC burden and disease severity in women with GS. As OF onset in GS females is nearly two decades earlier than in the general population, recognizing its association with greater BCC number and aggressiveness in young women has significant clinical implication in disease surveillance and early intervention. Given the role of the hedgehog signaling pathway in OF^5^ and the increased likelihood of women with OF being on systemic therapy in our study, it would be important to further investigate the potential benefits of early initiation of a hedgehog inhibitor in GS females with OF both to manage increased BCC burden and as a tissue-sparing therapy to preserve fertility.

## Conflicts of interest

None.

## Funding

None.

## Study approval

N/A

## Author contributions

TW - Methodology, Analysis, Data Curation, Writing - Original Draft, Review & Editing.

DP - Methodology, Analysis, Writing - Review & Editing.

JT - Conceptualization, Writing - Review & Editing.
